# Unusual Presentation of a Common Disease: Metastatic Germ Cell Tumor in the Brain

**DOI:** 10.7759/cureus.94141

**Published:** 2025-10-08

**Authors:** Anil Prasad, Radhika Narayan, Sree Devi Jakka, Amitabh Kumar Upadhyay, Manoj Kumar

**Affiliations:** 1 Pathology, Tata Main Hospital, Jamshedpur, IND; 2 Pathology, Manipal Tata Medical College, Jamshedpur, IND; 3 Medical Oncology, Tata Main Hospital, Jamshedpur, IND

**Keywords:** brain metastasis, case report, germ cell tumor, seminomatous, small round cell tumor

## Abstract

Brain metastasis (BM) as the first indication of a germ cell tumor (GCT) is quite rare. Typically, BMs are more frequently observed in patients who have relapsed GCTs, particularly those with non-seminomatous histology and extra-cerebral spread. When BMs are present at the initial diagnosis, they pose distinct diagnostic and treatment challenges. The detection of BMs at the time of diagnosis can suggest advanced disease. It may complicate the diagnostic process, particularly when there are no clear indications of a GCT. We reported a case of a 21-year-old male patient who experienced generalized seizures due to a mass lesion in the brain, which is uncommon. He presented to the hospital with a three-week history of right arm soft tissue swelling and a single episode of generalized seizures. A subtotal excision of the brain tumor was performed, and the patient was placed on ventilatory support. He was ultimately diagnosed with a mixed GCT. After receiving palliative whole-brain radiation therapy and four cycles of chemotherapy, the patient was kept on close follow-up but unfortunately succumbed later due to infectious complications. This case highlights the rare occurrence of BM at initial presentation in GCTs, underscoring the aggressiveness of these tumors. It also emphasizes the importance of maintaining a high index of suspicion, obtaining timely pathological diagnosis, and implementing appropriate management.

## Introduction

Originating from primordial germ cells, germ cell tumors (GCTs) are a broad spectrum of malignancies. They resemble embryonic development, exhibiting a range of morphological patterns and varying levels of differentiation. They are predominantly found in the gonads (testicles and ovaries), but also occur in extragonadal sites such as the anterior mediastinum, pineal gland, and brain. Testicular GCTs are considered rare neoplasms, accounting for only 1% of all male malignancies. Their occurrence is very low, with an incidence of approximately 3 to 11 cases per 100,000 men in Western countries. This figure may differ from one country to another [1]. They are of gonadal or extragonadal origin. Extragonadal GCTs (EGCTs) are malignant transformations of germ cells that are maldistributed during embryonic development or occur naturally to regulate immunological processes or extragonadal organ functions [2]. Seminoma and non-seminomatous subtypes are included. Histologically, 2-5% of malignant GCTs are of extragonadal origin [3]. They share many common features with gonadal GCTs, like midline locations, young age with male predominance, lung, liver, and bone metastasis, elevated tumor markers, and sensitivity to cisplatin-based therapy. However, EGCTs show increased tumor volume at first presentation and have a higher incidence of Klinefelter syndrome and hematological malignancies [4]. They share some biological similarities with GCTs but exhibit distinct epidemiological and histological features, as well as aggressive behavior with poor outcomes [5].

GCTs include seminomatous tumors (only classical seminoma) and non-seminomatous tumors, which comprise embryonal carcinoma (EC), teratoma (mature or immature), yolk sac tumor (YST), and choriocarcinoma. EGGCTs, which are constituted by two or more histological subtypes, are considered mixed subtypes.

Brain metastases (BMs) are observed in approximately one percent of patients diagnosed with disseminated GCT. A significant majority of these BM cases are associated with non-seminomatous GCT (NSGCT) [6].

We reported a rare case of mixed GCT in a young adult who first presented as a left arm tumor with BM and very poor performance status. The patient was kept on close follow-up but unfortunately succumbed later due to infectious complications.

## Case presentation

A 21-year-old male with an Eastern Cooperative Oncology Group performance status of 1 presented with complaints of right arm soft tissue swelling for two weeks, blurred vision in the left eye for ten days, a single episode of generalized seizure one day prior, and altered sensorium lasting for two hours. There was no history of fever, cough, or cold. The patient was admitted to the critical care unit and placed on ventilatory support. His past medical history was not significant except that a unilateral orchidectomy was performed three years ago due to a crushed and lacerated testis sustained in a road traffic accident; however, histopathology was not done. Clinical and ultrasound examination of the other testis revealed no abnormalities.

**Figure 1:**
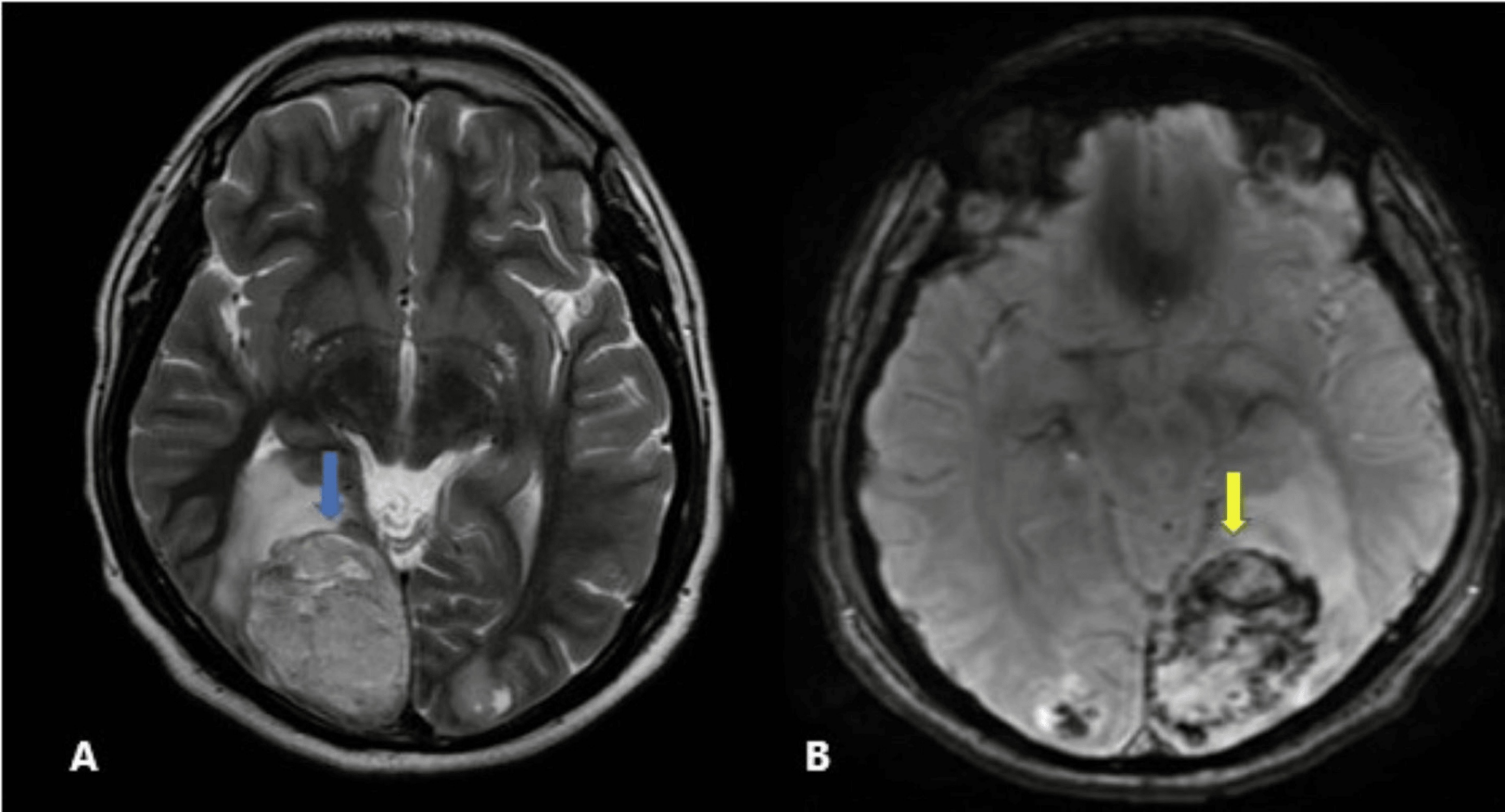
Radiological images (cranial MRI). (A) Axial T2 images showing a well-circumscribed heterogenous hyperintense lesion in the right posterior parietal lobe with surrounding edema (blue arrow). (B) GRE sequence demonstrating intralesional patchy hemorrhages (yellow arrow). MRI: magnetic resonance imaging, GRE: gradient echo

Investigations included a brain MRI conducted one week before admission; however, subsequent definitive action was deferred due to the patient's non-compliance. It showed multiple enhancing heterogeneous lesions, with the largest in the right parieto-occipital region (Figure 1A), and patchy intralesional hemorrhages (Figure 1B), suggestive of metastatic neoplastic pathology. Non-uniform enhancement of the lesions was revealed in post-contrast images.

**Figure 2:**
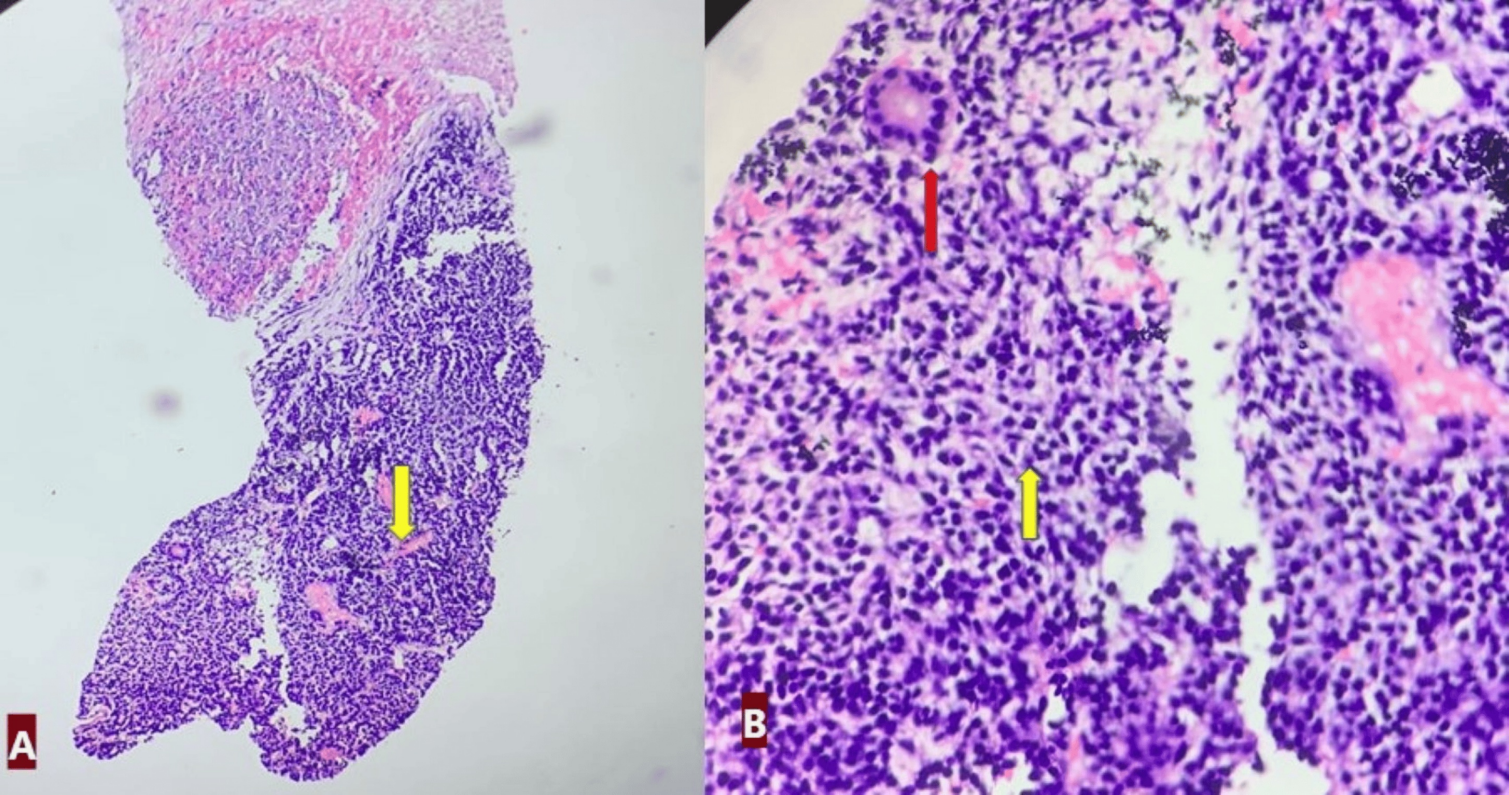
(A) Left arm lesion. Microphotograph showing small blue round cells in a sheet-like growth pattern (yellow arrow). (X100, H&E). B-cells are uniformly round to oval with hyperchromatic nuclei and scant eosinophilic cytoplasm (yellow arrow). Pseudorosettes are also seen (red arrow) (X400, H&E). H&E: hematoxylin and eosin

**Figure 3:**
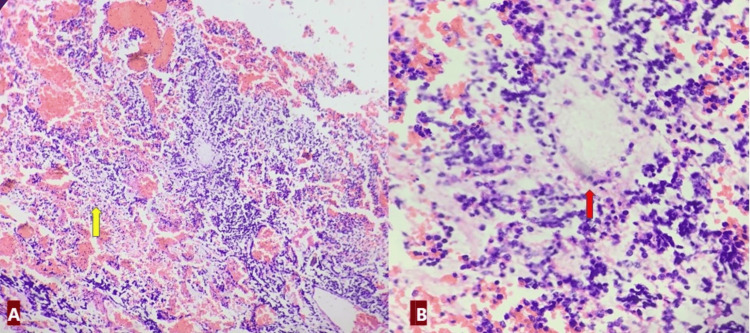
(A) Cranial lesion. Microphotograph showing blue round cells with hemorrhage and necrosis (yellow arrow). (X100, H&E). (B) Cells are loosely arranged with myxoid stroma (red arrow) (X400, H&E). H&E: hematoxylin and eosin

The hematological investigation was normal except for leucopenia. At the time, tumor markers were raised. Alpha fetoprotein was 10287 ng/ml (reference range: 0 ng/ml to 40 ng/ml), beta-human chorionic gonadotropin (β-hCG) was 6.36 mIU/ml (reference range for males: <2 mIU/mL), and LDH was 3767 U/mI (reference range: 45-245 U/L). Though nonspecific, tumor markers are helpful in the diagnosis and follow-up of EGCTs. Levels of human chorionic gonadotropin (i.e., β-hCG) are elevated in EC, choriocarcinoma, and in about 10% of cases of seminomas. Decompressive open craniectomy with subtotal excision of the brain tumor was done. Histopathological examination showed a small blue round cell tumor from soft tissue left in the left arm (Figure 2A-2B) and brain (Figure 3A-3B) lesion.

**Figure 4:**
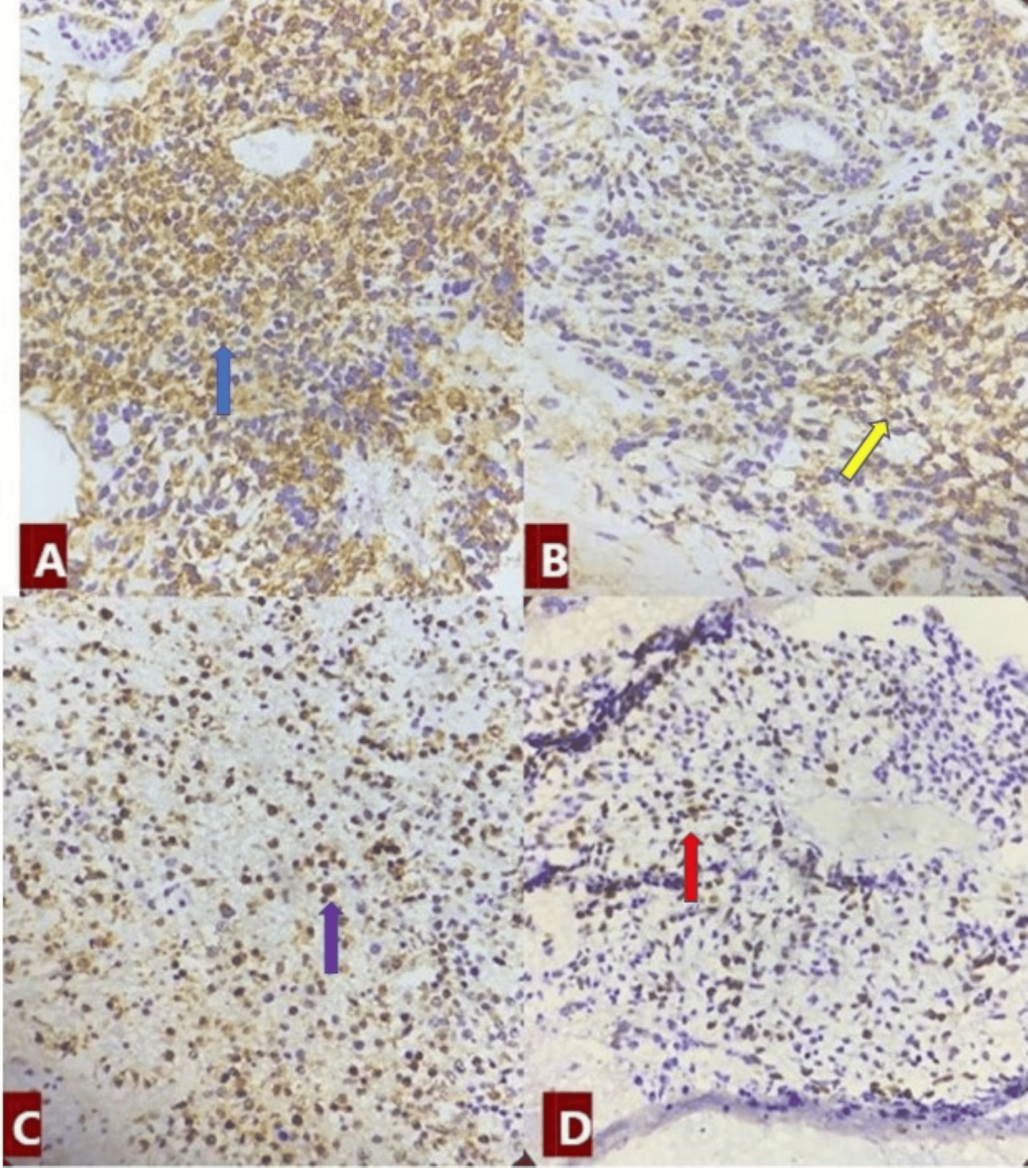
Positive immunohistochemical stains. (A) CK (blue arrow), (B) vimentin (yellow arrow), (C) CD99 (purple arrow), and (D) Ki67 index (red arrow). CK: pancytokeratin, CD: cluster differentiation

An immunohistochemical study showed the tumor was positive for CK (pancytokeratin) (Figure 4A), vimentin (Figure 4B), and CD99 (Figure 4C), with a high Ki67 index (Figure 4D). Tumor cells were also positive for SALL4, CD56, and CD117. However, GFAP, synaptophysin, P63, CK7/20, BCOR, EMA, NKX2.2, desmin, WT1, FLI1, SATB2, NUT, and SOXB2 were negative. These negative markers rule out tumors in the neural, salivary, sweat gland, and soft tissue areas. A negative NKX2.2 result excludes the diagnosis of Ewing sarcoma. The tumor was finally identified as a mixed GCT.

The patient got palliative whole-brain radiotherapy first, followed by palliative chemotherapy. He completed four cycles of the BEP regimen (bleomycin, etoposide, cisplatin) and has shown significant improvement in general condition. The patient was kept on close follow-up but succumbed later.

## Discussion

GCTs are a heterogeneous category of neoplasms originating from primordial germ cells, predominantly found in the gonads (testicles and ovaries), but also occurring in extragonadal sites such as the anterior mediastinum, pineal gland, and brain. Testicular GCTs are considered rare neoplasms, accounting for only 1% of all male malignancies. Fifty to seventy percent of all EGCTs found in adults originate from the primary mediastinum. BMs are found in 1-2% of all patients diagnosed with GCT and in approximately 10-15% of patients with advanced disease [7].

Metastatic central nervous system GCTs are rare malignant tumors characterized by their aggressive nature and unfavorable prognosis. The World Health Organization categorizes GCTs into two main types: seminomatous and non-seminomatous. The NSGCT consists of the YST, teratoma, embryonic tumor, and mixed GCT. Extragonadal NSGCT is rare, with a prevalence of 2-5% among GCTs [8].

BMs were predominantly symptomatic (53%), multiple (61.8%), and nearly always accompanied by metastatic sites in other locations (99.1%). The most common additional site was the lungs (94.3%), with frequent involvement of other extra-pulmonary visceral areas (42.1%) [9]. Males are more affected than their female counterparts with poor prognoses [10]. In GCTs, the presence of BMs at the time of initial diagnosis is a significant indicator of a poor prognosis. The preferred method for assessing soft tissue involvement and disease staging is MRI [6]. The relationship between a lesion and adjacent neurovascular structures is highlighted, which aids in planning biopsy and surgical resection.

Immunohistochemistry and molecular genetics are needed for the definitive diagnosis of the differentials. Microscopically, uniform small round cells are seen, arranged in sheets, pseudorosettes with round nuclei, fine stippled chromatin, inconspicuous nucleoli, and indistinct cytoplasm. Subgroups of tumors often show neuroectodermal differentiation (Homer-Wright pseudorosettes). Nuclear enlargement and prominent nucleoli are signs of atypical presentations.

Differentials include rhabdomyosarcoma, lymphoma, osteosarcoma, chondrosarcoma, synovial sarcoma, poorly differentiated carcinoma, high-grade neuroendocrine tumors, and metastatic tumors. The most commonly increased tumor markers are alpha-fetoprotein, β-hCG, and lactate dehydrogenase [11].

NSGCTs, especially those containing a component of YST, can exhibit increased alpha-fetoprotein levels and proportionally higher levels in advanced disease stages, whereas this is mostly expected in patients with pure seminoma. CK, PLAP, CD30, β-hCG, NANOG, and the SOX kit (CD117) are the most significant IHC markers [12].

The occurrence of BMs in patients with GCTs is uncommon, making their management a subject of ongoing debate and discussion [13]. Upfront high-dose chemotherapy is the best therapeutic option for these patients since the chance of survival using effective salvage chemotherapy in case of relapse is extremely low [13]. The standard chemotherapy regimen is cisplatin-based therapy, which includes three to four cycles of bleomycin, etoposide, and cisplatin. Specialized centers are needed to provide aggressive treatment with close follow-up for these patients [14].

Seminomatous GCTs are recognized for their high sensitivity to chemotherapy. At the same time, NSGCTs, which primarily give rise to BMs, have traditionally been considered resistant to radiation. The patient population is primarily young, and concerns about neurotoxicity weigh heavily in decisions regarding the recommendation of brain radiotherapy. Moreover, the aim of neurosurgery in managing BMs from GCTs is twofold: enhancing local brain control and eliminating any remaining teratoma, which is known to resist both chemotherapy and radiation [15].

Several studies have examined the cytogenetics of GCTs of both intragonadal and extragonadal origin. One or multiple copies of the short arm of chromosome 12p, accompanied by a loss of the long arm of chromosome 12 (12q), result in an isochromosome (i12p), which is commonly observed in most GCTs [16]. However, serial sections in human and murine embryos have not conclusively proven that germ cells have been misplaced outside the gonads. This case highlights the necessity for additional research to investigate the occurrence of brain involvement at the time of initial diagnosis and its subsequent outcomes.

The biological potential (aggressiveness of the tumor, including the ability to metastasize), the clinical course of treatment, and the long-term prognosis appear to vary based on anatomic location, age, sex, and, perhaps most importantly, the histologic subtypes, benign vs. malignant GCT, and the percentage composition of specific elements within mixed tumors. Efforts to characterize these clinical characteristics with imaging, serum tumor markers, and, more recently, circulating tumor DNA (ctDNA or cfDNA) are ongoing; challenges remain, including the heterogeneity and rarity of this tumor type, most likely necessitating multi-institutional (so-called basket) trials and meta-analyses to arrive at more definitive conclusions [17].

The lack of a histopathology report for the unilateral orchidectomy is a limitation of the study, as it is difficult to determine whether the tumor is a primary gonadal or de novo extragonadal germ cell.

## Conclusions

BM, as the first manifestation of a GCT, is quite rare. Typically, BMs are more frequently observed in patients who have relapsed GCTs, particularly those with non-seminomatous histology and extra-cerebral spread. When BMs are present at the initial diagnosis, they pose distinct diagnostic and treatment challenges. The presentation of this case highlights its unusual presentation, potential for misdiagnosis, rarity of occurrence, and aggressive nature. This case contributes to the growing body of literature on the rarity of initial brain presentation. It also highlights the need for more precise treatment guidelines, close follow-up, and further research to improve management and prognosis. A multidisciplinary approach, combined with adapted therapy, is preferred for a better understanding, treatment, and prognosis. Future trials may still evaluate new strategies specifically designed for patients with BMs from GCTs.
